# A novel method for radiotherapy patient identification using surface imaging

**DOI:** 10.1120/jacmp.v17i2.6066

**Published:** 2016-03-08

**Authors:** David B. Wiant, Quinton Verchick, Percy Gates, Caroline L. Vanderstraeten, Jacqueline M. Maurer, T. Lane Hayes, Han Liu, Benjamin J. Sintay

**Affiliations:** ^1^ Department of Radiation Oncology Cone Health Cancer Center Greensboro NC USA; ^2^ Department of Health Policy and Management University of North Carolina Chapel Hill NC USA; ^3^ Department of Physics Kenyon College Gambier OH USA

**Keywords:** patient identification, surface imaging, image‐guided radiotherapy, safety, AlignRT

## Abstract

Performing a procedure on the wrong patient or site is one of the greatest errors that can occur in medicine. The addition of automation has been shown to reduce errors in many processes. In this work we explore the use of an automated patient identification process using optical surface imaging for radiotherapy treatments. Surface imaging uses visible light to align the patient to a reference surface in the treatment room. It is possible to evaluate the similarity between a daily set‐up surface image and the reference image using distance to agreement between the points on the two surfaces. The higher the percentage overlapping points within a defined distance, the more similar the surfaces. This similarity metric was used to intercompare 16 left‐sided breast patients. The reference surface for each patient was compared to 10 daily treatment surfaces for the same patient, and 10 surfaces from each of the other 15 patients (for a total of 160 comparisons per patient), looking at the percent of points overlapping. For each patient, the minimum same‐patient similarity score was higher than the maximum different‐patient score. For the group as a whole a threshold was able to classify correct and incorrect patients with high levels of accuracy. A 10‐fold cross‐validation using linear discriminant analysis gave cross‐validation loss of 0.0074. An automated process using surface imaging is a feasible option to provide nonharmful daily patient identification verification using currently available technology.

PACS number(s): 87.53.Jw, 87.55.N‐, 87.55.Qr, 87.57.N‐, 87.63.L‐

## I. INTRODUCTION

A central tenet of health care is patient safety. Perhaps one of the greatest safety violations that can occur in radiation therapy is to treat the wrong patient or site. A number of simple safeguards, such as “time‐outs,” are employed across the medical community to check patient identity and treatment site.^1^, ^2^, ^3^ Time‐outs typically require staff members to verify patient name, birthday, treatment site, as well as compare the patient with a reference photograph prior to initiation of treatment. A time‐out system is simple and easy to implement and, in most cases, is quite effective. However, time‐out techniques are not foolproof, as a slight variation in process could lead to the wrong patient being treated. Consider a busy clinic with multiple staff members working at a treatment machine. One group of staff members verifies the patient information in the treatment room for breast patient “Jones,” while a second group opens the patient plan at the treatment control area outside the room for a different breast patient “Jones.” If the patient plan and identity are not carefully checked again prior to treatment a gross error would occur. Scenarios like this are not impossible, as the Pennsylvania Patient Safety Authority showed that 16% (n=4) of radiation therapy events reported from June 2004 through January 2009 were related to the wrong patient being treated.^(4)^

The Joint Commission recognizes that patient identification remains a critically important area of focus. For the past several years, including 2015, the Joint Commission has ranked “Identifying patients correctly” as the top goal in the Hospital National Patient Safety Goals.^(5)^ They suggest a similar approach to the time‐out with the recommendation to “use at least two ways to identify patients” to “make sure that each patient gets the correct medicine and treatment.”

Hendee and Herman^(6)^ discuss a hierarchy of short‐term effectiveness of patient hazard mitigation that places automation and computerization of tasks above policies, procedures, and checklists as ways to improve safety. Several different technologies have been tested that might allow for automation of patient identification. One such technology is a radiofrequency identification (RFID) system that uses implantable MOSFET radiation detectors to record radiation dose and emit unique RF signatures that allows the readout system to identify the patient.^(7)^ Another commercial system requires the patient to wear a RFID tag that can be tracked by sensors in treatment rooms to determine if the correct patient is on the table, and can automatically interface with record and verify systems.^(8)^ These techniques offer the possibility for robust, automated hard stops in the treatment process, but they may be expensive and invasive.

A better option may be to repurpose or expand existing treatment technologies to aid in patient identification. Recently, 3D surface imaging has emerged as a useful tool for patient setup and monitoring that is both noninvasive and nonionizing.^9^, ^10^, ^11^ The face and body shapes are unique to an individual patient. So in theory, a patient's surface image may be used to identify the patient without the need for dedicated equipment, invasive procedures, or additional radiation dose. In this work we examine the possibility of using surface images to uniquely identify breast patients.

## II. MATERIALS AND METHODS

**Figure 1:**
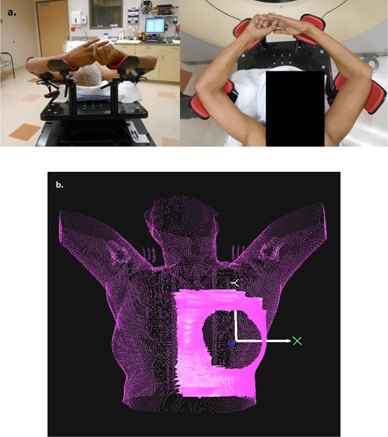
(a) Representative example of breast patient setup (b); representative example of the region of interest (ROI) used for initial patient alignment. The ROI is shown in solid pink; the reference surface is shown in dotted pink.

A series of 16 left‐breast radiotherapy patients with intact breast were retrospectively examined in this work. All patients were immobilized on a commercial breast board (QFix, Avondale, PA) at a 10° to 15° angle with both arms raised above their heads (Fig. 1). All of the patients underwent computed tomography scans on a Philips Big Bore Scanner (Philips Healthcare, Andover, MA) with nominal 3 mm slice thickness and the field of view set to include the patient and immobilization device. The patients were all contoured in the XiO (Elekta AB, Stockholm, Sweden) treatment planning system. The external, or body, contours were created using the auto‐threshold tool in XiO (all voxels with values >−850 HU were included in the contour). The typical interval between the treatment planning CT and the first treatment was 7‐10 days. The range of times between the first and last treatments was 22‐49 days.

All of the patients were set up and monitored daily with the AlignRT surface imaging system (VisionRT, London, UK). The AlignRT surface imaging system has been described in detail elsewhere.^9^, ^10^, ^11^ Briefly, AlignRT uses visible, nonionizing radiation to create a map of the patient surface in the treatment room that is updated several times per second. This treatment surface is aligned to a reference surface created from either the treatment planning process or a surface captured in the treatment room. Linear translations and rotations to bring the treatment surface into agreement with the reference surface are reported several times per second.

In this work, the treatment plans and contours were exported in DICOM format to AlignRT for treatment preparation. The body contours from the planning process were used to create reference surfaces in AlignRT. A surface generated from a DICOM formatted body contour will be referred to as a DICOM surface (DCMS). Regions of interest (ROI) for the AlignRT registration process were defined to include the ipsilateral chest wall and the base of the breast minus any pendulous breast tissue (Fig. 1).^12^, ^13^ The DCMS surfaces with these ROIs were used for daily patient setup. After initial setup, verification (or treatment) surfaces (VRTS) were acquired each day.

The DCMS and VRTS were retrospectively analyzed off‐line to evaluate the efficacy of surface imaging as a patient identification tool. Two types of comparisons were made between surfaces: 1) each DCMS was compared to 10 VRTS from the same patient, and 2) each DCMS was compared to 10 VRTS surfaces from each of the other patients (for a total of 150 comparisons with different patients’ VRTS).That is to say, Patient ‘A’ DCMS was compared to 10 randomly selected VRTS from Patient ‘A,’ 10 VRTS from Patient ‘B,’ 10 VRTS from Patient ‘C,’ 10 VRTS from Patient ‘D,’ et cetera. Comparisons were made using the AlignRT surface statistics tool. The surface statistics tool was used to evaluate “similarity” between surfaces based on the percentage of points on the verification surface falling within a set distance from the reference surface ROI.

**Figure 2:**
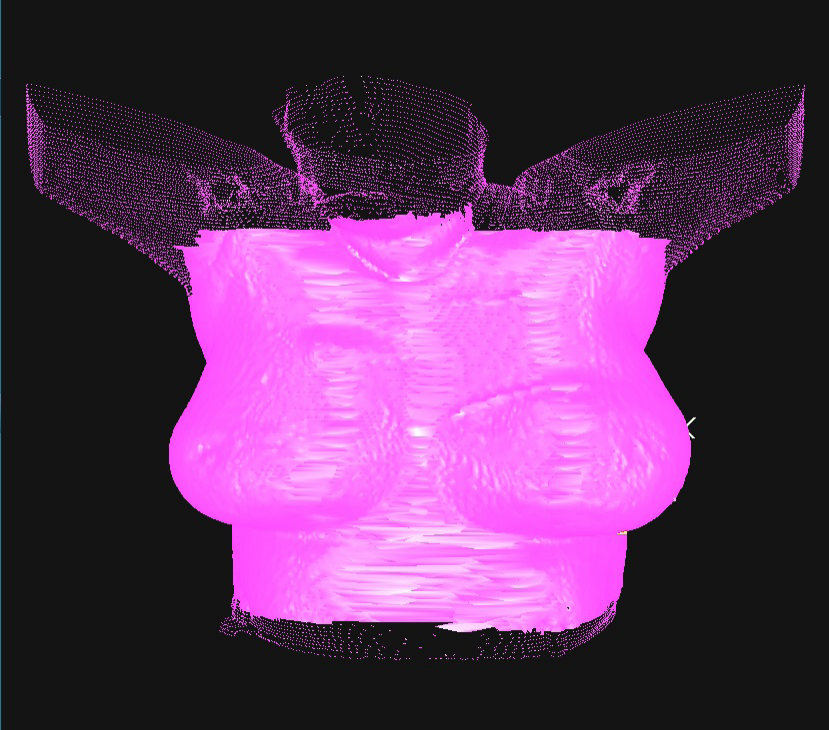
Example of the region of interest (ROI) used for similarity comparisons, where the ROI is shown in solid pink. The ROI was designed to cover both breasts, the neck, the chin, the abdomen, and the axilla.

The ROI used for the surface statistics comparisons differed from the setup ROI. Several iterations of the comparison ROI were performed to find a ROI that maximized the differences in the surface statistics similarity metric for comparisons between same patient and different patients. Examples of the ROIs used for the surface statistics comparisons are shown in Fig. 2. Prior to similarity evaluation the alignment of the comparison surface and the reference surface was optimized with six degrees of freedom using the proprietary AlignRT tool within the surface statistics toolbox. Distances to agreement thresholds of 3 mm and 5 mm for the surface statistics tool were used in this study. These thresholds are used to define a similarity score, which is the percentage of points on the verification image that map to points on the reference image within the distance to agreement threshold.

The similarity scores for each distance to agreement threshold were recorded for each comparison. Results of these measurements for each patient and for the group of patients as a whole were analyzed.

This work was performed with the approval of the Cone Health Institutional Review Board under protocol 1421.

## III. RESULTS

The mean age of the patients in the study was 67 years±11 years (range 46‐88 years). The average body mass index was $27\;\text{kg}/m^{2}\pm 6\;\text{kg}/m^{2}$ (range 20 kg / m^2^ to 39 kg / m^2^).

**Figure 3:**
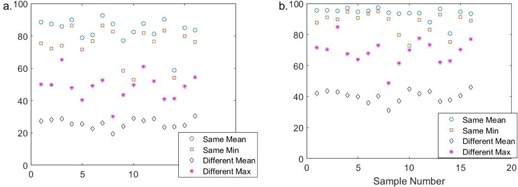
The mean and minimum same‐patient, and the mean and maximum different‐patient similarity scores are shown for each patient with (a) 3 mm and (b) 5 mm thresholds. For each patient the lowest same‐patient score does not overlap the highest different‐patient score.

The range of mean same patient similarity scores was 58.9% to 92.6% for the 3 mm threshold, and 80.6% to 97.5% for the 5 mm threshold. The minimum same‐patient similarity scores over all patients were 52.9% and 72.8% for the 3 mm and 5 mm thresholds, respectively. The maximum different‐patient scores over all comparisons were 65.2% and 85.0% for the 3 mm and 5 mm thresholds, respectively. The mean 3 mm and 5 mm scores for each patient are shown in Fig. 3. For all patients, at both 3 mm and 5 mm, the minimum same‐patient score was higher than the maximum different‐patient score, thus there was no overlap between same‐patient and different‐patient scores.

**Figure 4:**
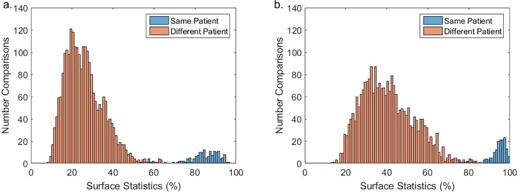
Histograms representing patient comparisons for (a) 3 mm and (b) 5 mm thresholds.

Histogram plots of every similarity score are shown in Fig. 4. The mean same‐patient similarity scores for the group were 83.6%±9.4% (range 52.9% to 96.8%) for the 3 mm threshold and 93.7%±5.3% (range 72.8% to 99.3%) for the 5 mm threshold. The mean different patient similarity scores were 25.9%±9.2% (range 8.6% to 65.2%) for the 3 mm threshold and 40.4%±12.6% (range 13.9% to 85.0%). Eleven out of 160 same‐patient scores were <65.2% (the maximum different patient score) for the 3 mm threshold, 13 same‐patient scores were <85.0% (the maximum different‐patient score) for the 5 mm threshold.

## IV. DISCUSSION

Previous work has looked at the use of automated patient recognition tools in a picture archiving and communication system to identify patients based on correlations of chest radiographs and showed the feasibility of this approach.^(14)^ Lamb et al.^(15)^ explored patient identification in the radiation oncology setting by means of comparing paired planar radiographic images to reference images in a 2D‐3D registration process. This technique was able to correctly identify patients using image pairs focused on the prostate, cranial, and thoracic/lumbar spine regions. This work was extended to evaluate 3D‐3D radiographic image registrations using an automated process by Jani et al.^(16)^ In this work they showed that registration similarity metrics could accurately identify patients and setup misalignments for head and neck, pelvis, and spine cases.

To the best of our knowledge this work represents the first attempts to apply this principle to the identification of radiation oncology patients using nonionizing surface imaging. On a per‐patient basis absolutely no overlap was observed between same‐patient scores versus different‐patient scores using the surface statistics similarity metrics with 3 mm and 5 mm thresholds. Minimal overlap was observed between same‐patient scores versus different‐patient scores for the group as a whole.

Based on these preliminary measurements, an automated process comparing daily VRTS to the reference DCMS could be imagined using a simple threshold, or cutoff value, to determine if the correct patient was being considered. Different thresholds could be set to optimize sensitivity (a wrong patient is identified correctly) and specificity (the correct patient is identified correctly). Based on the 3 mm threshold data, a threshold of 66% would yield 100% sensitivity (all incorrect patients identified) with 12 false‐positives (correct patients identified as being incorrect), or about 8% of the samples. A threshold of 55% would yield about 1% (2 of 160) false‐positives and 1% (16 of 2400) false‐negatives. A linear discriminant analysis with 10‐fold cross‐validation was performed using the MATLAB Statistics Toolbox software package (MathWorks Inc., Natick, MA) that yielded a misclassification probability of 0.0074. These numbers are comparable to what was seen by Lamb and Jani.

Lamb and colleagues^(15)^ used 2D‐3D radiographic image registrations to classify prostate and spine patients. In that work they found 0%‐10% of patients wrongly identified depending on threshold selection, and misclassification probabilities of 0.0045 and 0.014 for the prostate and spine, respectively. Jani et al.^(16)^ used 3D‐3D radiographic image registrations to classify head and neck, pelvis, and spine patients. They found <5% of patients wrongly identified for the evaluated sites depending on the technique, and misclassification probabilities of 0.0066, 0.0167, and 0 for the head and neck, pelvis, and spine, respectively. (This study looked at setup images generated by a Varian TrueBeam [Varian Medical Systems, Palo Alto, CA] and TomoTherapy [Accuray, Sunnyvale, CA]. The numbers shown here are for the TomoTherapy scans, which yielded slightly more accurate classifications.)

**Figure 5:**
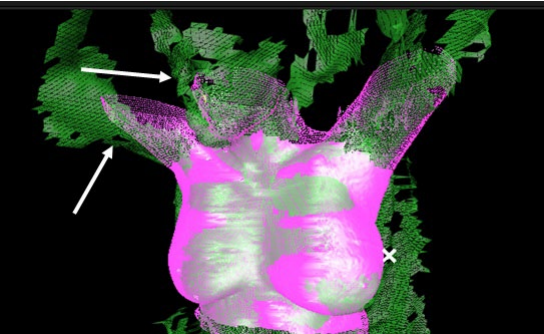
Example of a case with same‐patient similarity scores <60%. The solid pink surface is the reference region of interest (DCMS). The mesh green surface is the daily treatment surface (VRTS). The white arrows identify regions where the DCMS and VRTS show marked disagreement.

The false‐positive results described above came from three patients who had same patient similarity scores <60% for at least 1 fraction (one patient had eight scores <60%, one had two scores <60%, one had one score <60%). The ages and body mass indices of these patients were in the middle of the ranges for both metrics. Similarity scores may be affected by changes in body shape or posture, such as weight loss or unstable daily setup. The most likely reason for transient drops in similarity scores are daily setups that are poorly matched to the treatment planning setup. Consistently low similarity scores may indicate a combination of changes in body shape and inconsistent daily setup. Figure 5 shows an image of the patient that had eight same‐patient similarity scores <60%. Close inspection of the relationship of the VRTS to the DCMS shows that the patient's right arm and chin are raised towards the ceiling more in the VRTS than in the DCMS. This consistent change in body posture along with some inflammation likely led to the low similarity scores.

In the case of breast patients, false‐positives likely yield information about patient setup. A reasonable approach for surface imaging based patient identification might be to use a highly sensitive threshold and consider a value below the threshold indicative of an incorrect patient or a poor setup. Prospective analysis with patients or healthy volunteers might help to discern if there is a range of similarity scores that always indicates wrong patient, a range to indicate correct patient, and a range to indicate correct patient with poor setup.

In this work we observed no evidence of decreasing similarity scores over the course of treatment (which ranged from 22 days to 49 days). However, this was a relatively small sample size, so it cannot definitively discount gross changes in patient shape as a possible cause of false‐positive results. Shape changes along with poor setup should be considered when examining similarity scores that produce false‐positives.

All same‐patient‐to‐different‐patient comparisons were performed retrospectively. This did not allow for any patients to be physically setup to a different patient's ROI, which would likely be the case if the wrong patient were brought into a room for treatment. In theory, surface imaging guided setup with a different patient's ROI could make the agreement between the same patient and different patient surfaces increase (i.e., increase the similarity score). However, physically setting up to a different patient's ROI would likely have minimal impact on the results presented in this work. All of the patients in this study were left‐sided breast patients who were set up in the same commercial immobilization device with similar settings. Also, the treating therapists typically used surface imaging to move the patient with respect to machine isocenter, but did not change the posture of the patient based on surface imaging feedback, such that using a different patient's ROI would have had minimal effect on the patient posture. Use of surface imaging for patient identification in scenarios where the patient posture is grossly manipulated based on surface imaging information might warrant more consideration.

Analysis of larger patient groups to help optimize threshold levels and application to other disease sites is of interest for future work. Surface registrations are highly dependent on the amount of curvature (or topography) within the ROIs. Surfaces with distinct, smoothly varying features generally produce more accurate registrations than flatter surfaces. It would be of particular interest to evaluate how well a surface imaging based identification technique would perform on sites with different levels of curvature compared to the breast, such as the face or chest.

Analysis of larger patient groups to help optimize threshold levels and application to other disease sites is of interest for future work.

## V. CONCLUSION

Surface imaging based patient identification offers an exciting opportunity for daily, nonharmful automated verification of patient identity using existing technology. The feasibility of this approach has been shown for breast patients. Further work to validate an automated process and expand this to other disease sites is warranted.

## COPYRIGHT

This work is licensed under a Creative Commons Attribution 4.0 International License.
